# Case Report: Daily water monitoring provides an evidence-based guide for safe and effective use of diuretics—a longitudinal study

**DOI:** 10.3389/fcvm.2025.1625554

**Published:** 2025-07-30

**Authors:** Susie Cha, Douglas W. Wilmore

**Affiliations:** ^1^ Global Research and Development, Biospace, Inc., Cerritos, CA, United States; ^2^ Frank Sawyer Professor of Surgery Emeritus, Harvard Medical School, Boston, MA, United States

**Keywords:** heart failure, bioelectrical impedance analysis (BIA), fluid management, self-care, case report

## Abstract

Heart failure (HF) management commonly relies on diuretics, yet standard fixed-dose regimens fail to adjust for daily fluid fluctuations, often leading to suboptimal management. Current tools lack the real-time precision needed to adjust therapy in response to these fluctuations. Bioelectrical impedance analysis (BIA) is a tool that can potentially provide personalized guidance for adjusting diuretic therapy, but its daily clinical utility remains limited. We present the case of an 85-year-old patient with chronic HF who performed daily BIA measurements to guide diuretics administration over 1,201 days. The patient adjusted diuretic administration in response to rises in body weight and extracellular water to total body water (ECW/TBW) ratio, indicating fluid accumulation beyond his baseline variability. The patient maintained full adherence to daily BIA monitoring. Despite using diuretics on only 9.2% of the days, fluid balance remained stable with no changes in cardiac, renal, or electrolyte parameters. Fluid retention resolved within 24 h following tailored diuretic intervention. Additionally, daily BIA facilitated differentiation between fluid-driven from non-fluid-driven weight changes, improving informed decision-making. This case demonstrates that a daily BIA-guided, patient-led diuretic regimen was feasible and effective in maintaining fluid stability with minimal diuretic use. This approach may serve as a personalized self-care model for chronic HF management.

## 1 Introduction

Diuretics are central to managing congestive heart failure (HF) and fluid retention disorders (1). They provide acute relief and help maintain fluid balance, yet their long-term effects and optimal dosing strategies remain unclear (2). Current practice often relies on fixed daily doses, adjusted based on subjective indicators like weight changes, edema, or dyspnea (2, 3). However, this approach may not reflect a patient's true fluid status, potentially leading to excessive or unnecessary medication use, increased side effects, higher healthcare costs, and frequent electrolyte monitoring.

An individualized approach using objective fluid assessment could mitigate these risks. Bioimpedance analysis (BIA), a non-invasive technology that measures fluid volumes through tissue electrical properties, is one such method (4). BIA has shown promise in settings such as dialysis and critical care (5, 6), where accurate fluid assessment allows for tailored interventions that improve outcomes and reduce complications. Its effectiveness in these settings suggests it could refine diuretic dosing in HF management.

Advances in BIA technology have also improved its accessibility, making it suitable for home-based care. Patients could use BIA to monitor their fluid status and make informed decisions about diuretic use, fostering a proactive approach to fluid management.

Here, we present a case of a patient with chronic congestive HF who used daily BIA over a 3-year period to guide individualized, intermittent diuretic therapy. We retrospectively examine trends in fluid status, responses to medication, and factors influencing diuretic decisions. The case illustrates how daily, objective fluid assessment can support patient-specific, need-based diuretic management and may support a shift toward personalized treatment strategies in chronic HF management.

## 2 Case description

The patient was an 85-year-old male physician with a history of myocardial infarction in 2002, treated with stent placement and medication management. Due to an allergy to loop diuretics, he had been on chronic hydrochlorothiazide therapy. In 2020, he developed atrial fibrillation which reduced his ejection fraction from ∼40% to 25%–30%. At the time of the study, his daily medications included nebivolol, sacubitril/valsartan, empagliflozin, apixaban, simvastatin, and intermittent hydrochlorothiazide. This study was approved by the Planning Committee of Biospace, Inc., which provides institutional ethical oversight. The investigation conformed with the principles outlined in the Declaration of Helsinki (7), and informed consent was obtained.

**Figure 1:**
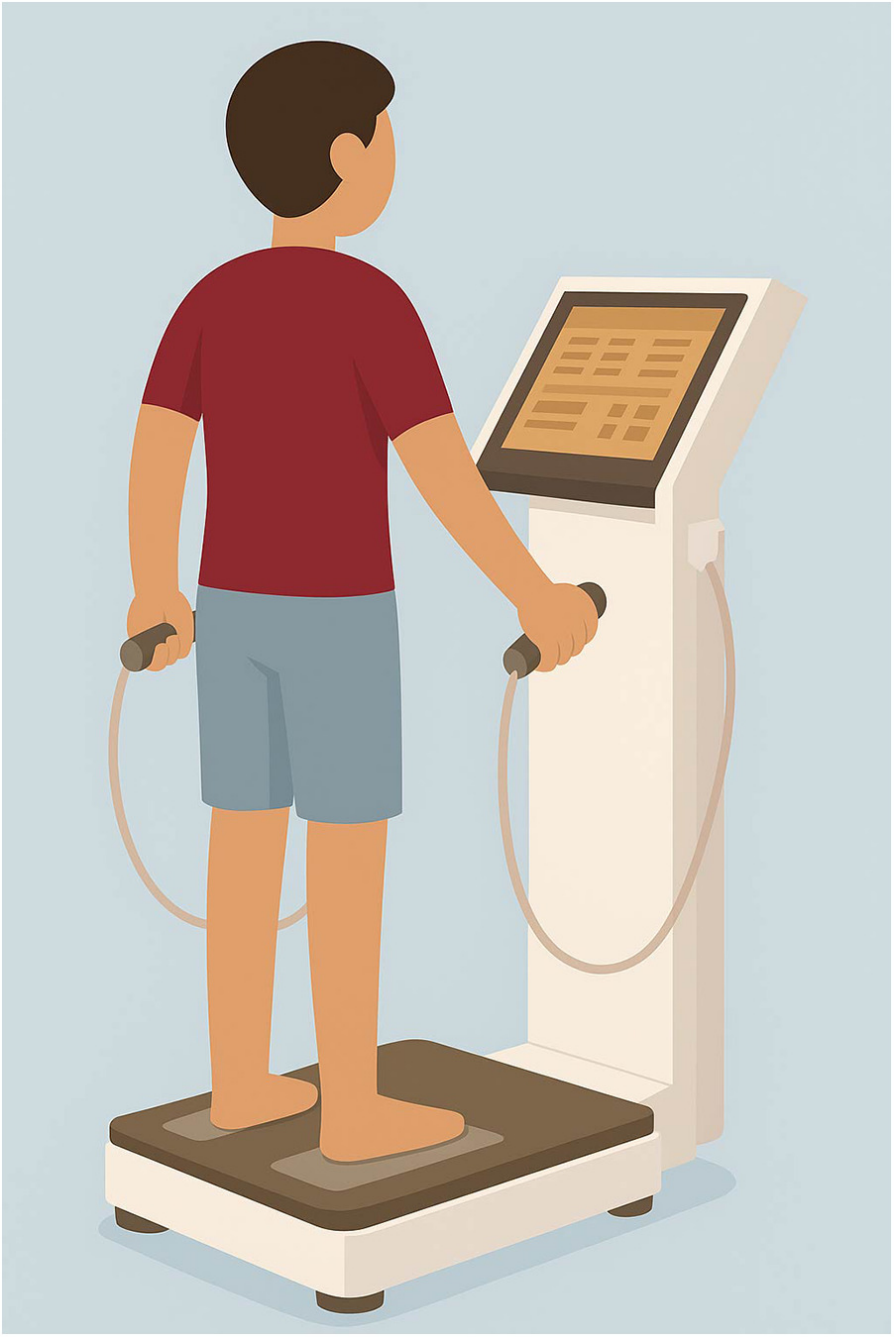
Illustration of a bioelectrical impedance analysis device in use. The measurement is performed with the subject standing barefoot on the scale and holding hand-grip electrodes while a low-level electrical current passes through the body to estimate body fluid. Created using ChatGPT (GPT-4).

Beginning in March 2020, the patient commenced daily BIA measurements using the InBody 770 (InBody Co., Ltd., Seoul, Korea), a non-invasive, stand-on device that estimates fluid status by measuring the body's resistance to a low-level electrical current passed between foot and hand electrodes (Figure 1). Each morning at 6:00 a.m., he adhered strictly to a standardized measurement protocol involving voiding, evacuating bowels, and avoiding food or drink, per manufacturer's guidelines. Measurements were taken without clothing to minimize variability.

Daily decisions about diuretic use were guided by trends in body weight and the extracellular water to total body water (ECW/TBW) ratio, both reviewed each morning. Although no fixed thresholds were used, the subject monitored these parameters for sustained upward shifts that might indicate of fluid retention. Diuretics were taken when such trends emerged. The relationship between these trends and diuretic use was later analyzed retrospectively to better understand the patterns preceding and following each intervention.

### 2.1 Data processing and statistical analysis

Time series data were detrended by computing a moving quantile baseline (45th percentile) using a 5-day sliding window, followed by Gaussian smoothing (*σ* = 1.5). This smoothed baseline was subtracted from the raw data to obtain detrended residuals. Outliers in the ECW/TBW ratio, defined as values >3 *σ* from the mean, were examined to assess fluid retention or imbalance and the safety of self-managed intermittent diuretic use.

Each diuretic administration was treated as a discrete event. Data were segmented into 7-day windows (3 days before and after use). Descriptive statistics (mean ± σ) were used to summarize weight and ECW/TBW across events. Temporal changes were assessed using repeated measures ANOVA and Tukey's *post hoc* test (*p* ≤ 0.05). Linear regression was used to test whether the ECW/TBW ratio on the day of diuretic administration (d0) predicted the magnitude of fluid reduction, defined as the absolute decrease in ECW/TBW ratio within the following 2 days.

The proportion of day-to-day weight changes due to fluid shifts was quantified to test whether acute weight fluctuations reflect fluid accumulation. Differences in weight and ECW/TBW ratios between consecutive days were standardized as *Z*-scores. Clustering analysis categorized these measures into two groups: weight changes driven by fluid shifts and those by non-fluid factors. Linear regression evaluated the relationship between day-to-day weight and ECW/TBW changes, with slope estimates compared between groups using two-sample *t*-tests (*p* ≤ 0.05).

All analyses were performed using MATLAB R2024a (MathWorks, Natick, MA).

### 2.2 Clinical findings

The subject maintained 100% adherence to the daily monitoring and decision-making framework over 1,201 consecutive days, from 19 March 2020, to 2 July 2023. Laboratory data remained unchanged (Table 1), and stable ECW/TBW ratios without outliers indicated no fluid retention or imbalance during this patient-driven diuretic regimen guided by daily BIA.

**Table 1:** Clinical, hemodynamic, and biochemical measurements at baseline and follow-up.

Parameter	Baseline Jan 2022	Follow-up March 2023
Hydrochlorothiazide dose, mg	50	50
Body weight, kg	76.2	77.5
Left ventricular ejection fraction, %	35	25
Mitral valve regurgitation, grade	Mild	Trace
Systolic BP (mmHg)	110	114
Diastolic BP (mmHg)	75	66
Heart rate, bpm	81	76
Hemoglobin, g/dl	16.0	15.2
Serum concentration		
BUN, mg/dl	20	15
Creatinine, mg/dl	0.83	0.98
Sodium, mmol/L	140	140
Potassium, mmol/L	3.6	4.8
eGFR, ml/min/1.73 m^2^	77	76

eGFR, estimated glomerular filtration rate; BUN, blood urea nitrogen.

**Figure 2:**
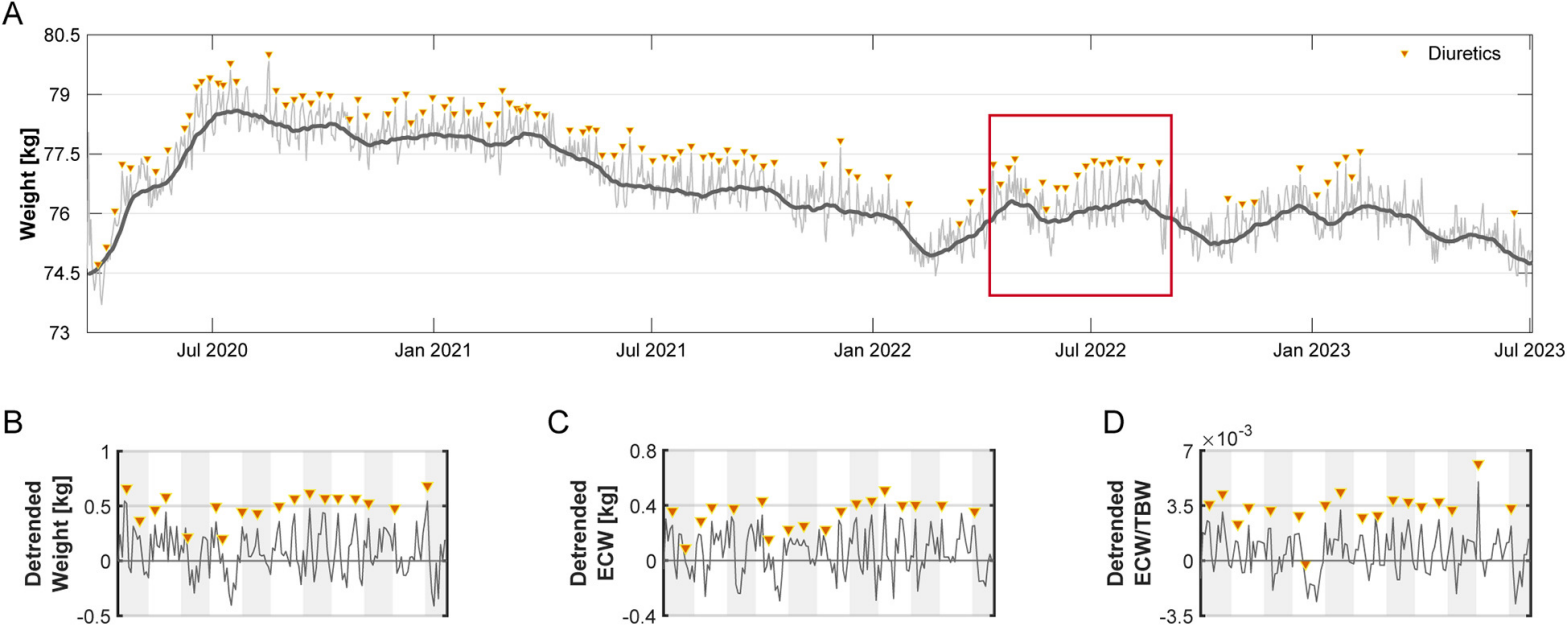
Long-term weight and fluid status over 1,201 days. **(A)** Daily weight (kg) measurements (thin line) recorded over 1,201 days, with a 30-day moving average (thick line) reflecting overall trends. Triangles mark days when diuretics were administered. **(B–D)** A 150-day segment (highlighted in Panel A) illustrating residuals (detrended) relative to baseline (set at 0) for **(B)** weight, **(C)** ECW, and **(D)** ECW/TBW ratio. Data reflect short-term deviations after long-term trend removal. Alternating shaded backgrounds indicate successive 14-day intervals.

Diuretics were used on 110 days (9.2%) at a median interval of 7 days (IQR: 6–10) (Figure 2A). The subject monitored body weight and ECW/TBW trends to guide diuretic use (Figures 2B–D).

### 2.3 Effect of diuretics on body water

**Figure 3:**
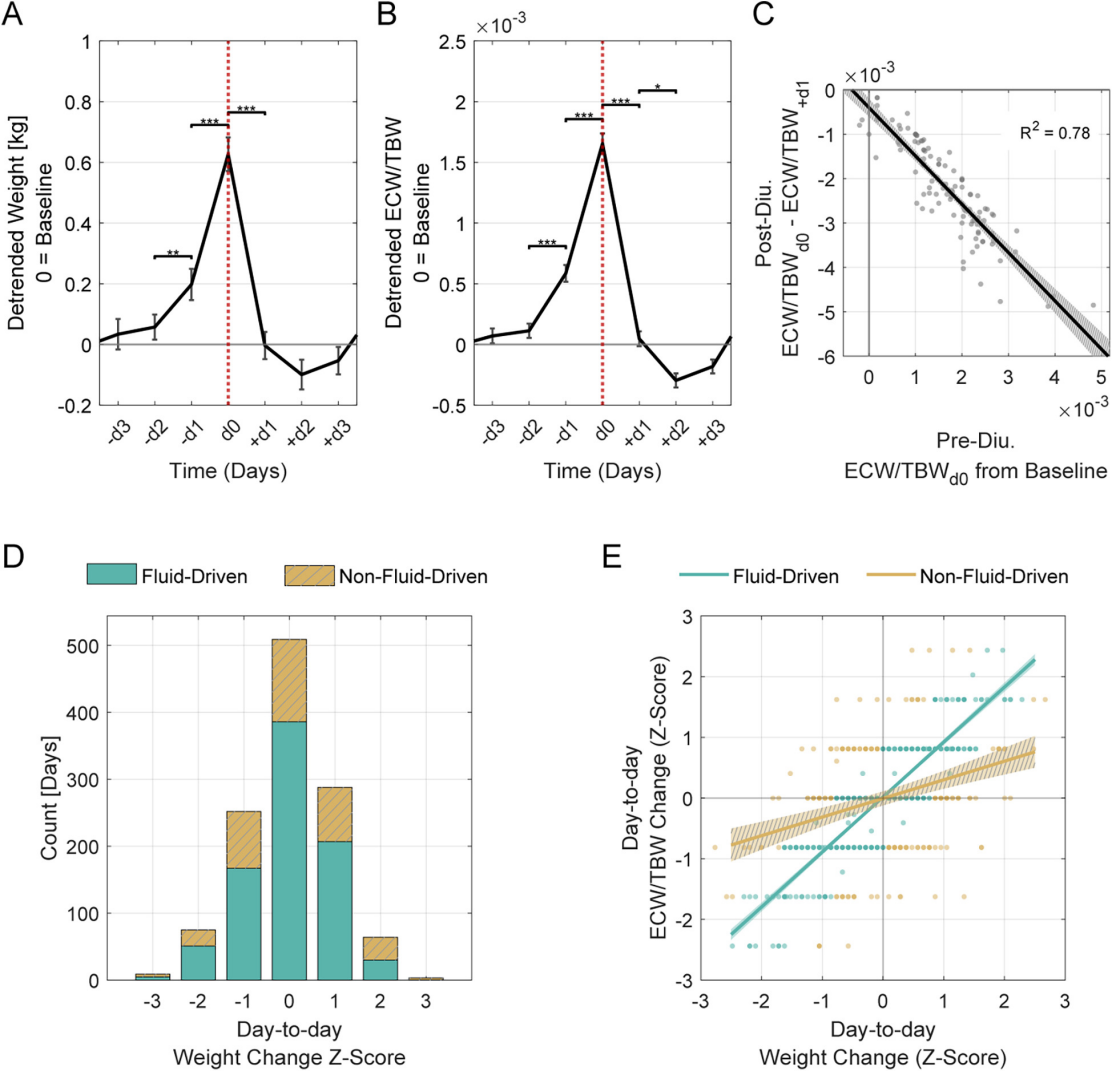
Acute diuretic effects and classification of day-to-day weight changes. **(A)** Weight and **(B)** ECW/TBW ratio changes over a 7-day window surrounding diuretic administration (d0, indicated by vertical dotted line). Data span from 3 days before (−d3) to 3 days after (+d3) administration. Solid lines represent the mean across all events, shown as residuals (detrended) relative to baseline (set at 0). Error bars indicate the standard error of the mean (SEM). Asterisks denote statistically significant differences between consecutive days (*p ≤ 0.05, **p ≤ 0.01, ***p ≤ 0.001). **(C)** Pre-diuretic fluid excess predicts post-diuretic fluid shifts. The *x*-axis shows ECW/TBW residuals at d0, indicating initial fluid excess, and the *y*-axis represents change in these residuals from d0 to + d1. A linear regression reveals a strong inverse association (*β* = −1.09, *R*^2^ = 0.79, *p* < 0.001). The shaded region represents the 95% confidence interval. **(D)** Histogram of day-to-day weight changes, classified as “Fluid-Driven” or “Non-Fluid-Driven” based on alignment between weight and ECW/TBW shifts. The *x*-axis represents day-to-day weight change, and the *y*-axis indicates the number of days classified in each group. Days were classified as “Fluid-Driven” if weight changes tracked closely with ECW/TBW ratio shifts, and “Non-Fluid-Driven” if the alignment was weak. **(E)** Weight-fluid relationships differ between groups. Day-to-day changes in weight and ECW/TBW are plotted as *z*-scores for each group (fluid-driven vs. non-fluid-driven), with linear fits and 95% confidence intervals (shaded regions). The fluid-driven group shows a stronger relationship between weight and ECW/TBW (*β* = 0.91, *R*^2^ = 0.78, *p* < 0.001) than the non-fluid-driven group (*β* = 0.31, *R*^2^ = 0.10, *p* < 0.001). A two-sample *t*-test comparing slopes confirms a significant difference between groups [t (388) = 226, *p* < 0.001].

Retrospective analysis showed diuretic administration consistently followed rises in these measures, particularly in the two days prior. Significant increases were observed from −d2 to −d1, with more pronounced rises from −d1 to d0 (Figures 3A,B; Table 2). On the day of administration, fluid parameters peaked above baseline (*p* < 0.01). Body weight increased by 0.63 kg, and the ECW/TBW ratio rose by 0.0017. Both measures returned to baseline within 24 h, indicating effective fluid removal. Linear regression showed that a higher ECW/TBW ratio on d0 predicted a greater reduction after diuretic administration, indicating that larger baseline fluid imbalances correspond to greater diuretic-induced fluid changes (Figure 3C).

**Table 2:** Weight, ECW, and ECW/TBW ratio relative to baseline on the day of diuretic administration (d0), 1 day before (−d1), and 1 day after (+d1).

Parameter	Mean, σ (95% CI)		
	Day of diuretics d0	Pre-diuretic −d1	Post-diuretic +d1
Weight, kg	0.63 ± 0.26 (0.58, 0.68)	0.20 ± 0.24 (0.15, 0.24)	0.00 ± 0.21 (−0.04, 0.04)
ECW, kg	0.36 ± 0.20 (0.32, 0.39)	0.11 ± 0.18 (0.08, 0.15)	−0.01 ± 0.16 (−0.04, 0.02)
ECW/TBW (×10^−3^)	1.66 ± 0.87 (1.49, 1.82)	0.59 ± 0.73 (0.45, 0.72)	0.05 ± 0.64 (−0.08, 0.17)

Data are presented as mean ± standard deviation (σ) and 95% confidence intervals (CI). d0, day of diuretic administration; −d1, one day before diuretic administration; +d1, one day after diuretic administration; ECW, extracellular water; TBW, total body water.

### 2.4 Relationship between weight gain and fluid accumulation

Weight gain is commonly used as an indicator of fluid retention but can be influenced by non-fluid factors. Clustering day-to-day weight and ECW/TBW changes revealed two groups: “Fluid-Driven” (*n* = 846), where weight changes were primarily fluid-related, and “Non-Fluid-Driven” (*n* = 354), where fluid shifts were minimal. A silhouette score of 0.74 confirmed strong cluster separation (Figure 3D).

In the “Fluid-Driven” group, weight changes strongly correlated with ECW/TBW ratio shifts, while the “Non-Fluid-Driven” group showed a weaker relationship (Figure 3E). The difference in slopes between these groups was statistically significant.

We further analyzed the clustering results to determine the proportions of “Fluid-Driven” and “Non-Fluid-Driven” groups among larger weight gains, which are more likely to reflect fluid retention. Among moderate weight gains (*Z* ≥ 1; + 0.48 kg; *n* = 149), 65% were Fluid-Driven and 35% Non-Fluid-Driven. Among larger gains (*Z* ≥ 2; + 0.95 kg; *n* = 25), Non-Fluid-Driven predominated (76%) over Fluid-Driven (24%).

## 3 Discussion

This case study examines a patient-led, BIA-guided approach to diuretic management. The patient, an 85-year-old male with congestive HF who was unable to tolerate loop diuretics due to allergy, demonstrated the feasibility of a responsive, data-driven alternative to fixed dosing. Over the course of 36 months, he maintained stable cardiac, renal, and electrolyte balance while using diuretics on only 9.2% of study days. Daily BIA monitoring enabled precise fluid management, reducing routine blood tests and healthcare costs.

Traditionally, weight monitoring is widely used to track fluid retention in HF; however, weight tracking alone is an unreliable indicator of fluid retention in HF monitoring (2, 8). Our results showed that only 65% of moderate and 24% of larger weight gains were due to fluid retention. This suggests that day-to-day weight changes are often influenced by other factors, such as diet and bowel movements, which challenges the assumption that significant weight gain is primarily due to fluid retention.

BIA provides a rapid, non-invasive method for assessing fluid compartments (4–6) and offers a practical alternative to traditional tracer techniques (e.g., deuterium oxide and bromide) that are impractical for routine use. In our study, the ECW/TBW ratio reliably identified fluid retention and informed treatment decisions, while segmental BIA of the lower extremities improved early detection by identifying fluid accumulation before clinical signs, such as visible swelling, appeared.

Regular, standardized measurements established a baseline for monitoring fluid changes over time. Consistent timing and pre-measurement routines minimized variability, ensuring that observed changes reflected true fluid shifts rather than normal fluctuations. Daily assessments revealed distinct fluid retention patterns, and retrospective analysis showed that diuretics were administered when the ECW/TBW ratio and weight exceeded baseline fluctuations by 1.34σ. Since daily fluctuations vary among individuals, establishing a threshold (e.g., 1σ above baseline) may provide an objective criterion for early detection of abnormal fluid shifts and support timely, personalized diuretic interventions. Post-diuretic BIA readings confirmed that fluid levels returned to baseline, providing objective evidence of effective decongestion and mitigating concerns about undertreatment and delayed exacerbation that are often associated with intermittent diuretic therapy.

Long-term adherence in chronic disease management is often challenging, yet in this study, adherence remained high over 3 years. The simplicity and efficiency of the protocol, with its 1-min measurement, likely supported sustained use by minimizing daily burden. Additionally, real-time, actionable feedback reinforced adherence by giving the subject a tangible sense of control over his treatment, instilling confidence, and deepening engagement in self-care.

The findings of this study are based on a single participant and should be interpreted with caution. Several factors likely contributed to the successful use of BIA for diuretic management in this case. The subject was a physician who was able to make informed, clinically sound decisions about diuretic use based on his medical knowledge and experience. He also demonstrated strong adherence to daily monitoring, maintained consistent dietary and activity patterns, and had access to regular clinical oversight to support accurate interpretation of BIA data and safe diuretic adjustments. However, these ideal conditions may not reflect those typically found in the broader patient population.

Wider adoption of BIA-guided diuretic management will require a clearly defined clinical framework that includes standardized BIA interpretation protocols, decision support tools, patient education, and daily professional oversight. For patients with complex or unstable conditions, BIA should be integrated into a broader care strategy that includes regular electrolyte monitoring and personalized treatment plans to ensure safety and optimize outcomes. Further research involving larger, more diverse cohorts and comparisons with standard care approaches is needed to validate these preliminary findings and assess their generalizability across different healthcare settings.

Nonetheless, this case underscores the potential value of daily BIA-guided diuretic management as a proactive, individualized approach to chronic HF management. By enabling patients to objectively assess their fluid status and respond with precision, this method could substantially reduce unnecessary medication exposure and healthcare burdens while maintaining treatment efficacy. Further clinical exploration and validation in larger patient populations are warranted to evaluate broader applicability and benefits in routine clinical practice.

## 4 Patient perspective

Managing HF without loop diuretics created ongoing uncertainty about fluid control. Daily weight measurements alone were inadequate and left me uncertain about treatment decisions. Incorporating daily BIA monitoring reduced this uncertainty by providing an objective fluid measure. Over time, clear patterns emerged that gave me confidence in knowing when diuretics were needed. This simple daily routine became an easy, reassuring habit that made a complex condition feel more manageable.

## Data Availability

The raw data supporting the conclusions of this article will be made available by the authors upon reasonable request.
